# Enzymes in Biofuels Production

**DOI:** 10.4061/2011/658263

**Published:** 2011-11-20

**Authors:** Sulaiman Al-Zuhair, K. B. Ramachandran, Mohamed Farid, Mohamed Kheireddine Aroua, Praveen Vadlani, Subramanian Ramakrishnan, Lucia Gardossi

**Affiliations:** ^1^Department of Chemical and Petroleum Engineering, UAEU, Al-Ain 17555, UAE; ^2^Department of Biotechnology, Indian Institute of Technology Madras, Chennai 600036, India; ^3^Department of Chemical and Materials Engineering, The University of Auckland, Auckland 1142, New Zealand; ^4^Department of Chemical Engineering, University of Malaysia, 50603 Kuala Lumpur, Malaysia; ^5^Department of Grain Science and Industry, Kansas State University, Manhattan, KS 66506, USA; ^6^Department of Chemical and Biomedical Engineering, Florida A&M University, Tallahassee, FL 32310-6046, USA; ^7^Dipartimento di Scienze Farmaceutiche, Universita' Degli Studi di Trieste, 34127 Trieste, Italy

With the inevitable depletion of the nonrenewable resources of fossil fuels and due to their favorable environmental features, biofuels promise to be the preferred fuels of tomorrow. They can displace petroleum fuels and, in many countries, reduce the dependence on imported fuel. Biofuels, derived from biomass conversion, such as biodiesel, bioethanol, biohydrogen, and biogas, are sustainable and renewable sources of energy, which are also considered CO_2_ neutral. In addition, burning biofuels results in reduced levels of particulates, carbon oxides and sulfur oxides, emissions compared to fissile fuels. 

To respond to the increased demand for biofuels, advanced biochemical processes using enzymes are being developed, which are gaining increased global attention. Research in this field aims at improving efficiency, and reducing negative environmental impacts, of production processes, in addition to enhancing the quality of the produced biofuels. Enzymes have been employed to overcome the drawbacks associated with the use of conventional chemical catalysts. For example, biodiesel production by enzymatic catalyzed processes is less energy intensive and more environmental friendly compared to its production by conventional alkaline catalyzed processes. In addition, the biocatalyst allows using unrefined feedstock, including waste oil, readily without the need to separate the free fatty acids that may be present in large amounts in the feedstock. Another example is the use of enzymes for the hydrolysis of cellulose to produce fermentable sugars for bioethanol production. The utility cost of enzymatic hydrolysis is much lower compared to the alternative methods of acidic hydrolysis because it is carried out at mild conditions and does not require subsequent treatment step. 

There are several obstacles, however, facing the use of enzymes as catalysts for biofuels production, most importantly is their high costs. Therefore, repeated use of the enzymes is essential from the economic point of view, which can be achieved by using them in immobilized form. In a continuous process using immobilized enzyme, the operational stability, the exhaustion of enzyme activity, and inhibition by reactants and/or products play vital roles. The use of membrane bioreactors for the enzymatic processing is increasingly becoming more attractive, as such systems allow continuous separation of products and prevent enzyme inhibition. 

Research attention is also focused on genetic engineering in enzymes production. Recently, genes of various enzymes have successfully been cloned, and more genes are promised to be cloned rapidly in the coming years. The use of recombinant DNA technology to produce large quantities of recombinant enzymes will help lower the enzymes costs. In addition, protein engineering will help to create novel enzyme proteins that are more resistant and highly thermo-stable. The introduction of a new generation of cheap enzymes, with enhanced activities and resilience, should change the economic balance in favor of enzyme use.

It gives me great pleasure to present to you this special issue. The issue covers both basic and applied aspects of using enzymes in the production of various types of biofuels. Articles published present different aspects of current and potential involvement of enzymes in biofuel production.

